# Myocardial Bridge and Angiotomography of the Coronary Arteries: Perfusion under Pharmacological Stress

**DOI:** 10.5935/abc.20170021

**Published:** 2017-06

**Authors:** Wilter dos Santos Ker, Daniel Gama Neves, Alair Sarmet A. A. Damas, Cláudio Tinoco Mesquita, Marcelo Souto Nacif

**Affiliations:** 1 Hospital Universitário Antônio Pedro (HUAP) - Universidade Federal Fluminense (UFF), Niterói - Brazil; 2 Hospital Pró-Cardíaco, Rio de Janeiro, RJ- Brazil; 3 Complexo Hospitalar de Niterói (CHN), Niterói - Brazil

**Keywords:** Myocardial Bridge, Myocardial Ischemia, Perfusion, Radionuclide Imaging, Computed Tomography, Coronary Artery Disease

## Introduction

The myocardial bridge is one of the most prevalent congenital anomalies that involve the coronary circulation, and its incidence in the general population is high, affecting from 23 to 55% in necropsy studies.^1^ The impairment of the anterior descending artery is more frequent, on its proximal 2/3.^1^ In most patients, the myocardial bridges do not cause symptoms, because in order to have ischemia, there must be an imbalance between supply and consumption of oxygen. The superficial bridges, with small or slender muscle band, are the most common ones, and they may account for 75% of the cases, with average length of 1.5 cm and usually without causing symptoms. In approximately 24% of the cases, we observed deep myocardial bridges, with thicker muscle band.^1,2^

Atherosclerosis is the most common cause of ischemic heart disease. However, other causes for ischemia are frequent, and among them, we highlight the myocardial bridge, which may provoke typical or atypicalchest angina,acute myocardial infarction and sudden death.^3-5^

Angiotomography of the coronary arteries is an increasingly important diagnosis technique when assessing the myocardial bridge, with high spatial and temporal resolution. Thisnoninvasive imaging technique is a very useful tool for locating and defining the morphology of the myocardial bridge.^6^

## Objectives

We describe the case of a female patient with myocardial ischemia detected through myocardial scintigraphy on which the determining mechanism for presence of the perfusion alteration was a bridge diagnosed by the angiotomography of the coronary arteries, which also confirmed the of the perfusion defect by evaluating resting perfusion images and those under pharmacological stress.

## Case Report

Female patient, 52 years old, presenting atypical chest pain, witha 26.5 body mass index, diabetic, hypertensive, dyslipidemic, usingASA, ARBS, Insulin, Metformin. She was forwarded to the nuclear medicine sector with a Myocardial Scintigraphy request for ischemia survey.

**Figure 1:**
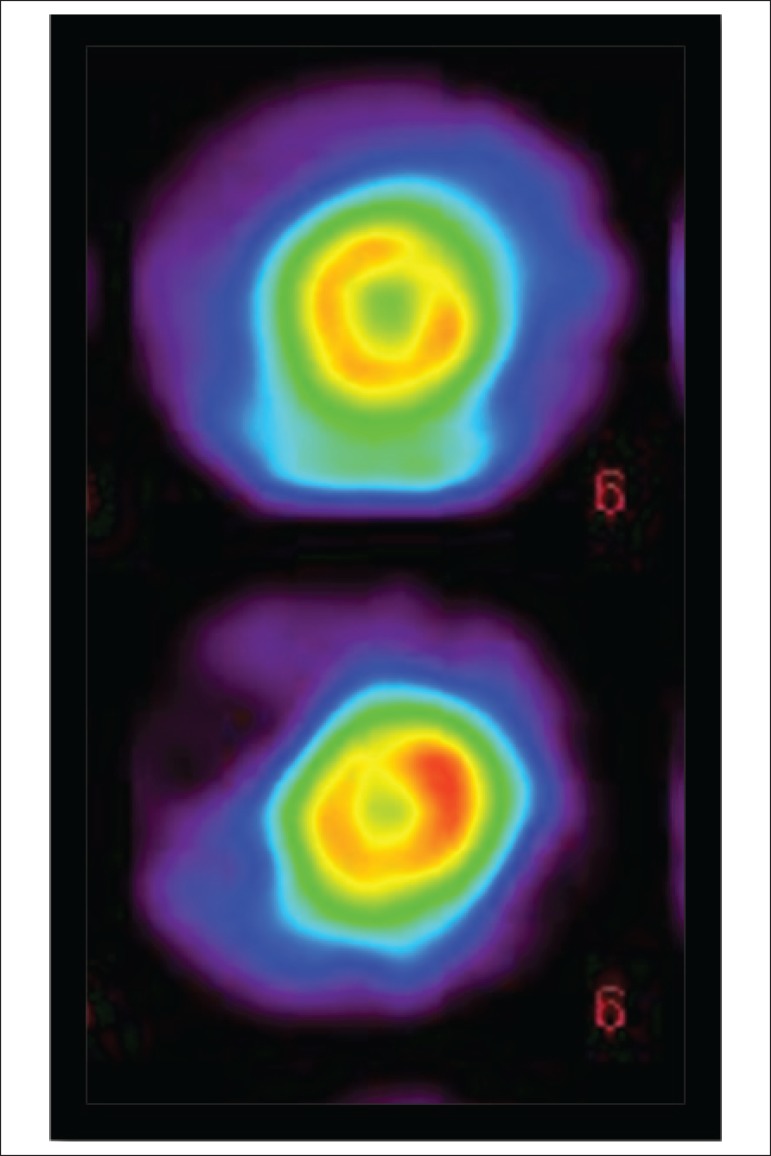
Myocardial scintigraphy with 2-methoxy isobutyl isonitrile -99mTc (sestamibi-99mTc) using protocol (rest-stress), with a 25 mCi dosein each step. The scintigraphic images reveal hypoperfusion in anteroapical and lateral-apical segments of the left ventricle in the post-stress images, with complete improvement of the uptake in relation to the rest images.

**Figure 2:**
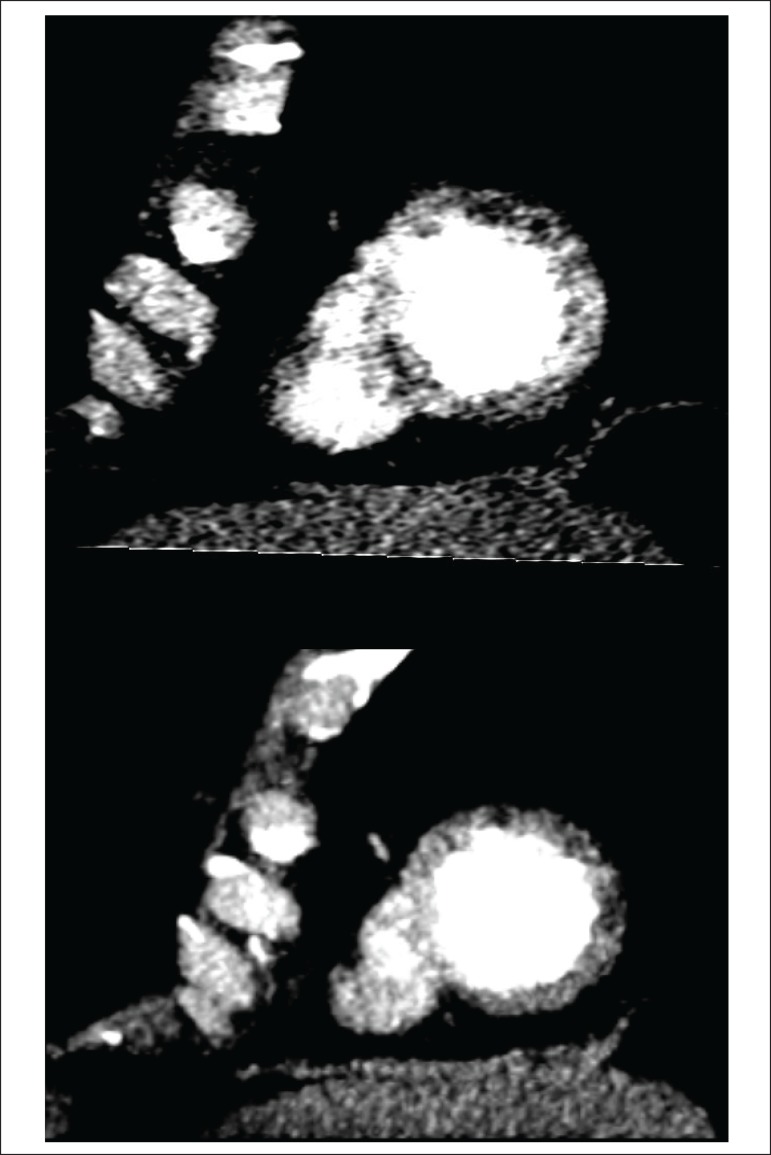
64-channel Cardiac Computed Tomography, effort and rest stage, reveals perfusion defect in the anteroapical and lateral-apical segments of the left ventricle in the post-stress images with normal perfusion in rest.

The patient was invited to participate in the research project, approved by the ethics committee no. 392,966, which aims to compare the perfusion findings of the nuclear medicine exam to those from the angiotomography of the coronary arteriesat rest and under stress. The patient performed a specific myocardial scintigraphy procedure (Figure 1) on a 1-collimator Gamma Camera device (Millennium MPR, GE) and acomputed tomography scan of 64 detectors (Brilliance, Philips), to evaluate the calcium score, myocardial perfusion at rest and under stress associated with coronary anatomical evaluation. The stress acquisition was conducted following dipyridamole infusion at a dose of 0.56 mg/kg, in 4 minutes. On the sixth minute, 25 mCi of 2-methoxyl-isobuthyl-isonitrile-99 m Tc (sestamibi-99mTc) was administered. In the same minute, the perfusion images under pharmacological stress by angiotomography (Figure 2) were acquired, with infusion of iodinated contrast at a 70 ml dose under a 5 ml per second flow rate. The myocardial perfusion scintigraphy images, stress stage, were acquired 30 to 90 minutes after the administration of the radiopharmaceutical.

**Figure 3:**
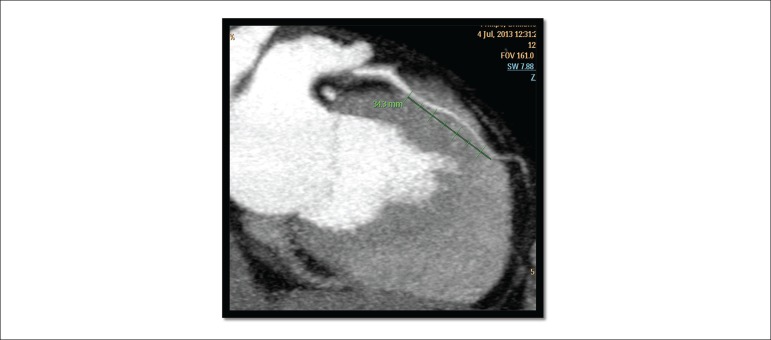
Myocardial CT angiography showing presence of a 34.3 mm myocardial bridge, in the projection of the anterior descending artery, responsible for the area of perfusion defect in the anteroapical and lateral-apical segments of the left ventricle, described in the myocardial scintigraphy and computed tomography.

The stress scintigraphic images demonstrated reversible perfusion defectswithin the territory of the anterior descending artery. Theperfusion computerized tomography confirmed the presence of perfusion defects and did not evidence a presence of atherosclerotic lesion in coronary arteries. A significant myocardial bridge constricting the anterior descending artery was diagnosed by the angiotomography of coronary arteries (Figure 3), configuring the most probable mechanism for the observed perfusion defects.

## Discussion

The myocardial bridge still shows various challenges to the clinical practice, because it may occur in patients on which the atherosclerotic disease is uncommon. This leads, in several situations, to failure to reach a diagnosis due to the low pretest probability of these patients. As it is a known factor for myocardial ischemia, the myocardial bridge may hamper the clinical correlation when linked to the atherosclerotic disease, complicating the planning of the best therapeutic management and monitoring of these patients. In the case of myocardial bridges, the mechanism responsible for the symptoms is uncertain and controversial. The irrigation of the vascular myocardium occurs almost exclusively during diastoles, and the bridge reduces the light of the artery, in most cases, only during systoles. As such, it is not easy to explain the physiopathology of myocardial ischemia.^7^

Among the various hypothesis, we may mention the distortion of the intramyocardial artery during systoles, provoking myocardial ischemia. The presence of symptoms only in individuals whose myocardial bridges are long and deep is favorable to this hypothesis. This mechanism could be aggravated when the oxygen consumption by the myocardium increased. The appearance of coronary spasm in the anterior descending artery in its intramyocardium path after intracoronary injection of acetylcholine appears to be another hypothesis, suggesting endothelial dysfunction located in that segment. This seems to be the reason for the symptoms to appear only in the fourth or fifth decade of live, a time on which alterations to the vascular tonus occur.^8,9^ Theendothelial injury is also implied in the formation of thrombi at the proximal region of the coronary bridge.^10^

The diagnosis through clinical examination is difficult precisely because the symptoms are virtually identical to those of the atherosclerotic artery disease. Functional studies validating the effect of the myocardial bridge on the myocardial blood flow demonstrate that its restriction occurs both during the systoles as well as in diastoles, and that there is a link between reversible myocardial ischemia shown by scintigraphy or by the positron emission tomography.^11^ Thevasodilator pharmacological stress may not be linked to ischemia because there is no increase in the coronary contractility and subsequent systolic compression.^11^ The angiotomography (angio-CT) of the coronaries is an exam that allows viewing the cardiac anatomy, especially that of the coronary arteries, in addition to analyzing the vessel walls, the presence of plaques and the diameter and course of the arteries. Barros and collaborators demonstrated that angio-CT is highly accurate in the morphological evaluation of the myocardial bridge, allowing a noninvasive approach of its localization, length and depth, as well as of the presence of associated atherosclerosis.^12^ The association of the coronary angio-CT with the functional study of the myocardial perfusion under stress with dipyridamole allows for a better definition of the physiological and clinical significance of this condition, as observedin the present case, where there is functional significance.^12^

In most cases of myocardial bridge, the prognostic is good, after the start of medication use, but there are reports of sudden death in in young people when exercising. The drug treatment is able to control the symptoms in the vast majority of cases, using beta-blockers and antagonists of calcium channels, providing better filling of the diseased coronary during diastoles, reducing the heart rate at rest and during efforts. Nitrates may aggravate the anginal symptoms and the ischemia when used in patients with myocardial bridge, because this drug promotes the reduction of the venous return and blood pressure with consequent adrenergic stimulation, increasing the systolic constriction of the myocardial band on the coronary artery. Currently, the drug treatment is the preferential for the myocardial bridges, because, in proper doses, it may control the angina episodes in most of the patients.^12,13^

We believe that the angiotomography of the coronary arteries, when used with protocol at rest and under pharmacological stress, may gather useful information for handling the patient withprecordial pain without significant obstructive coronary disease, be it diagnosed by catheterization or any other method of characterization of ischemia, as demonstrated by scintigraphy in the instant case.
